# Preparation, Characterization, and Insecticidal Activity of Avermectin-Grafted-Carboxymethyl Chitosan

**DOI:** 10.1155/2016/9805675

**Published:** 2016-04-24

**Authors:** Yan Li, Yukun Qin, Song Liu, Ronge Xing, Huahua Yu, Kecheng Li, Pengcheng Li

**Affiliations:** ^1^Institute of Oceanology, Chinese Academy of Sciences, No. 7 Nanhai Road, Qingdao 266071, China; ^2^University of Chinese Academy of Sciences, Beijing 100049, China; ^3^Key Laboratory of Experimental Marine Biology, Institute of Oceanology, Chinese Academy of Sciences, No. 7 Nanhai Road, Qingdao 266071, China

## Abstract

Avermectin-grafted-N,O-carboxymethyl chitosan (NOCC) derivative was obtained by esterification reaction using dicyclohexylcarbodiimide (DCC) as dehydrating agent and 4-methylaminopyridine as catalyst. The structures of the conjugate were confirmed by FT-IR, ^1^H NMR, and XRD. Insecticidal activities against armyworms, carmine spider mites, black bean aphids, and brown plant hoppers were investigated at concentrations ranging from 0.16 to 1000 mg/L. At the concentration of 1000 mg/L and 500 mg/L, the lethal rate was 100%. Good insecticidal activity at 4 mg/L was still shown, especially against the black bean aphids and brown plant hoppers. Moreover, the photostability of the conjugate was evaluated and showed an apparent improvement. At 300 mins, the residual rate of the conjugate was 11.22%, much higher than 0.2% of the avermectin technical material. The conjugate we developed showed potential for further study and application in crop protection.

## 1. Introduction

Avermectin (AVM), a family of macrocyclic lactones derived from* Streptomyces avermitilis* with potent anthelmintic and insecticidal properties, has been widely used to control parasites and pests of human being, animals, and crops [1]. Particularly, it occupies great share in pesticides market due to its broad spectrum and high efficiency [2]. Now, the commercial product of avermectin mainly consists of liquid (Emulsifiable Concentrate) and granular formulations. It means lots of emulsifying agents and organic solvents would be used to maintain the performance of these types of avermectin formulations [3], although they can offer a wide distribution of the active compound throughout the soil profile. And it will undoubtedly pose a threat to human health and the environment [4]. Moreover, avermectin is sensitive to light and thus photolysis will occur under the exposure of light, which may lead to its decomposition [5].

Chitosan, a kind of natural cationic polysaccharides, consisted of *β*-1,4-linked glucosamine with various N-acetylglucosamine residues [6]. It is a natural, renewable resource and exhibits a lot of special properties like biocompatibility, biodegradability, and bioactivity. However, chitosan also shows some demerits: it is only soluble in dilute acids but insoluble in water and most organic solvents, which greatly limits its application in many areas. Thus, there have been a lot of methods to improve its solubility [7]. One of the approaches is the chemical modification of chitosan. Among the various modified products, carboxymethyl chitosan is a kind of water-soluble derivative [8]. Compared with chitosan, carboxymethyl chitosan shows enhanced biological properties, such as antimicrobial, antioxidant, and apoptosis inhibitory activities [9]. It has been intensively studied and considered as a very promising substitution for chitosan in many applications, including agriculture, medical treatment, and food industry [10].

Recently, the synthesis and property study of chitosan conjugates have received increased attention. Different kinds of molecules are grafted onto chitosan chains by chemical bond [11]. The complexation of chitosan and metal ions enhanced its antifungal activity [12, 13]. The conjugation by grafting of antioxidant molecules onto chitosan improves the antioxidant activity [14]. However, little attention has been paid to the improvement of chitosan insecticidal activity by the conjugation of insecticidal molecules [15–18].

In order to improve the insecticidal activity of chitosan and the photostability of avermectin technical material at the same time, we did our research on the conjugation of chitosan and avermectin technical material. In our study, avermectin was grafted onto the carboxymethyl chitosan polymer chains for the first time by esterification employing DCC as dehydrating agent and 4-methylaminopyridine as catalyst. The synthesized avermectin-grafted-NOCC was characterized by Fourier transform infrared (FT-IR), nuclear magnetic resonance (NMR) spectroscopy, and X-ray diffraction (XRD) to confirm the conjugation. The insecticidal activities and photostability of the grafted copolymers were also determined.

## 2. Experiment

### 2.1. Materials and Reagents

Chitosan was purchased from Qingdao Yunzhou Biochemical Corp. with an average molecular weight of 1060 kDa and deacetylation degree of 85.3%. Avermectin technical material was purchased from Shijiazhuang Ruitian Biochemical Corp. Dicyclohexylcarbodiimide (DCC) and 4-methylaminopyridine (DMAP) were purchased from Xiya Chemical Reagent Co., Ltd. Isopropanol, monochloroacetic acid, methanol, acetic acid, dichloromethane, pyridine, ethanol, dimethylformamide, and Tween 80 were purchased from Sinopharm Chemical Reagent Co., Ltd., and were all of analytical grade. Acetonitrile was purchased from Merck Drugs & Biotechnology and was of chromatographic grade.

### 2.2. Analytical Methods

Fourier transform infrared (FT-IR) spectra were investigated on a Thermo Scientific Nicolet iS10 FT-IR spectrometer ranging in the 4000–400 cm^−1^ regions with attenuated total reflection intelligent components. ^1^H NMR (nuclear magnetic resonance) spectra were investigated on a JEOL JNM-ECP600 spectrometer; solvents were CD_3_COOD and D_2_O. The grafting ratio of avermectin-grafted-NOCC was determined by an Agilent 1260 HPLC (Agilent Technologies, USA) equipped with a UV-detector. Chromatography was performed on C18 reversed-phase column.

### 2.3. Synthesis of N,O-Carboxymethyl Chitosan (NOCC)

Purified chitosan (3 g) was dispersed in 65 mL of isopropanol. After 20 minutes of magnetic stirring at room temperature, 20.4 g of aqueous NaOH (40%) and 14.4 g of monochloroacetic acid/isopropanol solution (1 : 1 m/m) were added to the suspension. The reaction proceeded to 4 h at a temperature of 60°C. After the reaction was finished, product was filtered, added into methanol (150 mL), and neutralized with acetic acid (1 M). The final product was washed by ethanol (80%) and dried at room temperature [19].

### 2.4. Synthesis of Avermectin-Grafted-NOCC

NOCC (2 g) and dicyclohexylcarbodiimide (1 g) and 4-methylaminopyridine (0.1 g) were mixed in a solution of 40 mL dichloromethane and pyridine (v/v = 3 : 1); then mechanical stirring lasted for 6 h at room temperature. When the activation process was done, avermectin technical material (4 g) dissolved in 10 mL dichloromethane was slowly added into the reaction system in drops. The reaction time was set as 12 h. The reaction liquid was filtered. The raw product was washed with dimethylformamide and ethanol to remove unreacted AVM and by-product dicyclohexylurea (DCU). Finally the product was dried at 60°C. Finally the conjugate of avermectin-grafted-NOCC (AVM-g-NOCC) was obtained.

### 2.5. Determination of Grafting Ratio of Avermectin-Grafted-NOCC

0.1 g AVM-g-NOCC was added into acetonitrile solution (100 mL, pH 4.0). Hydrolysis lasted 2 hours to extricate all the avermectin from the conjugate. Then, the solution was diluted to 10 times for detection. The determination of AVM-g-NOCC was measured with Agilent 1260 HPLC (Agilent Technologies, USA) equipped with a UV-detector. Chromatography was performed on C18 reversed-phase column, using H_2_O/acetonitrile (15 : 85) solution as mobile phases at a flow rate of 1.0 mL/min with column temperature at 30°C. The testing wave length was set to 245 nm [20].

### 2.6. Insecticidal Activity Assay

Samples were prepared with aqueous solution with 0.1% Tween 80 and diluted to 7 concentrations including 2 high concentrations and 5 low concentrations for insecticidal activity assay.

The insecticidal activity for armyworms was carried out by leaf dipping method [21]. Corn leaves were naturally air-dried and then placed in the petri dish after being fully immersed in the sample solution. 10 third-instar larvae were raised at the temperature of 24–27°C in each dish for 2 days. Then the dead numbers were noted.

The insecticidal activities for carmine spider mites, black bean aphids, and brown plant hoppers were carried out by spray method [22]. Broad bean leaves with carmine spider mites and brown plant hoppers and rice seedlings with black bean aphids were used to spray sample solutions with Potter spray tower; then we culture and observe the dead amount in prescriptive room after 48 hours, respectively.

### 2.7. Photostability Assay

Avermectin-grafted-NOCC and avermectin technical material aqueous solution with a concentration of 0.1 mg/mL were treated by irradiation of UV 365 nm wavelength light. At testing time point, 1 mL solution of all samples was analyzed in ultraviolet spectrophotometer of 245 nm to calculate the surplus ratio of the conjugate and avermectin technical material. The testing time points were set to 10, 30, 50, 70, 100, 150, 200, 250, 300, and 360 min.

## 3. Results and Discussion

### 3.1. Preparation of N,O-Carboxymethyl Chitosan and Avermectin-Grafted-NOCC

**Figure 1:**
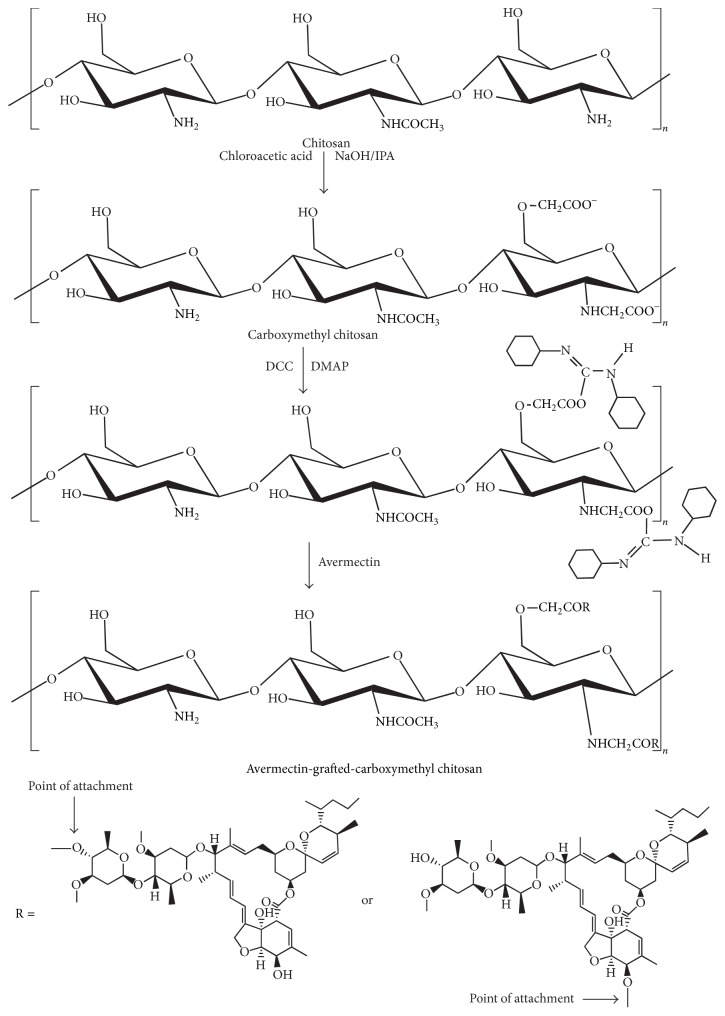
The proposed mechanisms for the synthesis of avermectin-grafted-NOCC: synthesis of NOCC and conjugation of avermectin onto NOCC.

In our research, avermectin-grafted-NOCC was synthesized successfully. The esterification reaction was conducted by using DCC as dehydrating agent and 4-methylaminopyridine as catalyst. The mechanism of grafting reaction of avermectin with N,O-carboxymethyl chitosan was shown in Figure 1. Firstly, the synthesis of N,O-carboxymethyl chitosan was conducted by mixing chitosan and monochloroacetic acid in the solution of isopropyl alcohol and NaOH. The degree of carboxylation was 42.65%. Then, the nitrogen-atom DCC donated electron to the oxygen atom in the carboxyl group of NOCC. The complexation of DCC and NOCC initiated the polymerization. Then, the hydroxyl of avermectin was connected to the carbonyl group of the intermediate and rearrangement formed was identified. The conjugate and by-product dicyclohexylurea (DCU) were achieved in the very end. The reaction product of avermectin-grafted-NOCC copolymer was a kind of water-soluble white powder. The graft ratio measured by HPLC was 3.66 mg/g.

### 3.2. Characterization of N,O-Carboxymethyl Chitosan and Avermectin-Grafted-NOCC

#### 3.2.1. FT-IR Spectra

**Figure 2:**
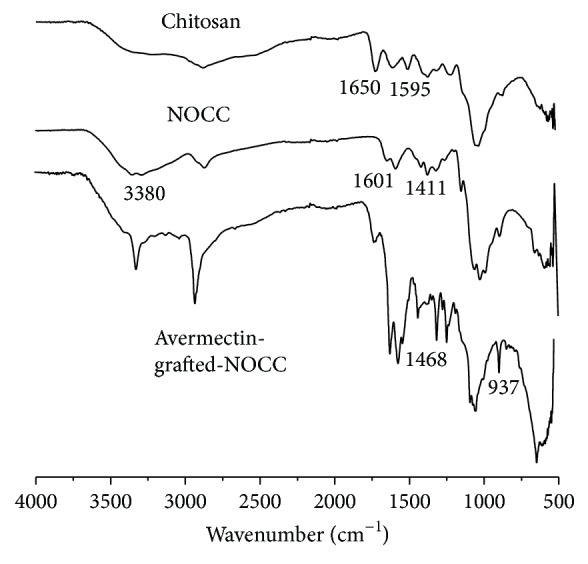
FT-IR spectra of chitosan, NOCC, and avermectin-g-NOCC.

Figure 2 shows FT-IR spectra of chitosan, NOCC, and avermectin-grafted-NOCC. For chitosan, the absorption peak of N-H bending was assigned to the band at 1595 cm^−1^. The absorption peaks of the C-O stretching, N-H bending, and C-N stretching of N-acetyl groups were at 1650, 1550, and 1320 cm^−1^, respectively. For NOCC, the bands at 1601 and 1411 cm^−1^ could be attributed to the stretch vibration of COO^−^ [23]. Additionally, the absorption peaks of symmetric stretching of the C-O-C appeared at 1156 cm^−1^, 1076 cm^−1^, and 1029 cm^−1^. For avermectin, the absorption peaks at 935 cm^−1^ were the stretching vibration of ethylenic bond. The bands at 1470 cm^−1^ were attributed to C-H bending vibration. Compared with NOCC, new bands at 1468 cm^−1^ and 937 cm^−1^ were observed. All of the results above indicated that avermectin-grafted-NOCC had been synthesized successfully [15].

#### 3.2.2. ^1^H NMR Spectrum

**Figure 3:**
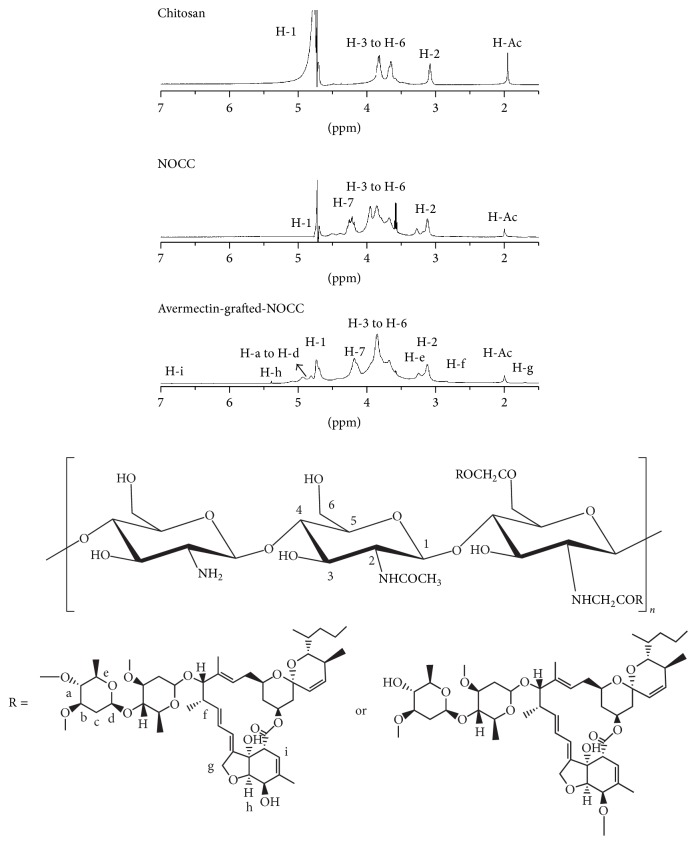
^1^H spectrum of chitosan, NOCC, and avermectin-g-NOCC.

The ^1^H NMR spectra of NOCC, avermectin grafted avermectin are shown in Figure 3. Further characterization of chitosan, NOCC, and NOCC grafted copolymers was performed by using ^1^H NMR. As shown in Figure 3, a series of peaks belonging to chitosan was detected: H-1 signal was the single peak at 4.4 ppm, H-2 peak was at 2.9 ppm, and peaks of H-3 to H-6 were the multiple peaks from 3.3 ppm to 3.7 ppm. The signal of protons of N-acetyl glucosamine groups was at 1.8 ppm, showing a single peak [8]. For NOCC, the peaks became broader and illegible than the initial chitosan. The characteristic absorption peak of -CH_2_-COO- groups was at 4.4 ppm [24]. Compared with NOCC, a series of new peaks of avermectin-g-NOCC was observed. New peaks at 7.9 ppm, 7.5 ppm, 6.8 ppm, 5.4 ppm, 5.1 ppm, 4.9 ppm, 4.8 ppm, 3.8 ppm, 3.1 ppm, 1.7 ppm, and 1.19–1.22 ppm were assigned to the protons of avermectin such as aliphatic protons, vinyl protons, and cyclic protons, which were marked in Figure 3 separately. Consistent with the FT-IR results above, the structure of avermectin-grafted-NOCC was further confirmed by ^1^H-NMR.

#### 3.2.3. Crystallographic Structures

**Figure 4:**
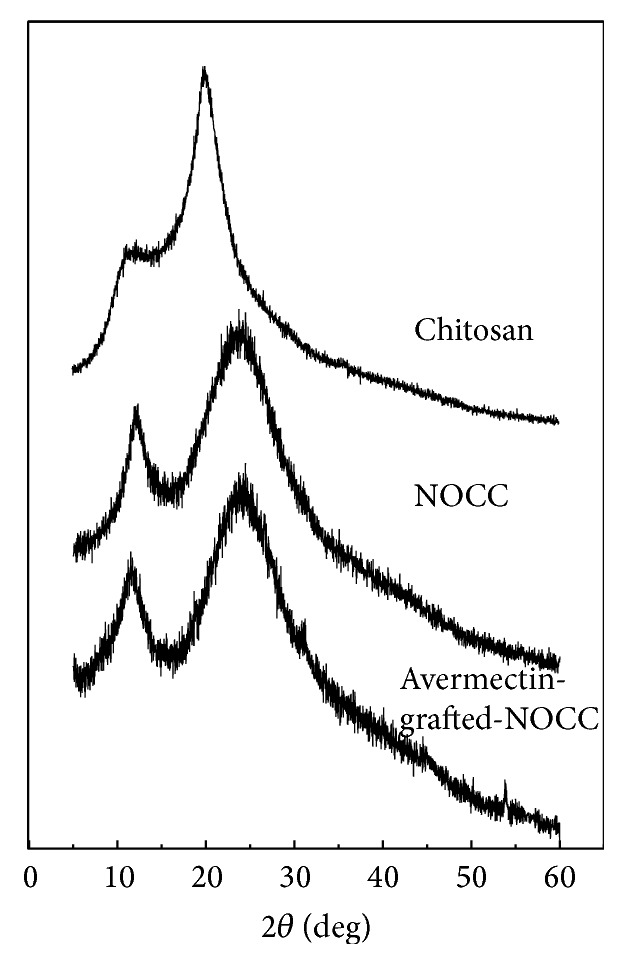
XRD patterns of chitosan, NOCC, and avermectin-g-NOCC.

Figure 4 showed the crystallographic patterns of chitosan, NOCC, and avermectin-grafted-NOCC. As shown in the XRD chart, crystalline reflections of chitosan were observed. The diffraction peaks were at 12° and 20°. For NOCC, the characteristic peaks were at 2*θ* = 12° and 24°; meanwhile, they both became broader. The introduction of carboxymethyl group caused obvious decrease in crystallinity, leading to the increase of water solubility. Avermectin-grafted-NOCC copolymer exhibited peaks at 2*θ* = 11.5° and 23.6°. The crystal structure of NOCC was changed by the conjugation. The modification of NOCC altered the macromolecular conformation. Compared with the semicrystalline structure of chitosan, the structure of avermectin-grafted-NOCC copolymer was amorphous.

### 3.3. Insecticidal Activity of Avermectin-Grafted-NOCC

Insecticidal properties of avermectin-grafted-NOCC against armyworm, carmine spider mites, black bean aphids, and brown plant hoppers were investigated at 2 high concentrations and 5 low concentrations ranged from 1000 to 0.16 mg/L.

It has been proved that different factors such as molecular weight, graft ratio, and concentration could influence the effect of chitosan and its derivatives. In this study, samples were prepared with aqueous solution containing 0.1% polysorbate 80, avoiding any organic solvent. In this way, the toxicity of organic solvents on targets was eliminated. This is also the expected condition for the application of the conjugate in the future applications. No addition of organic solvents would reduce the pollution of the environment and benefit the food security.

**Figure 5:**
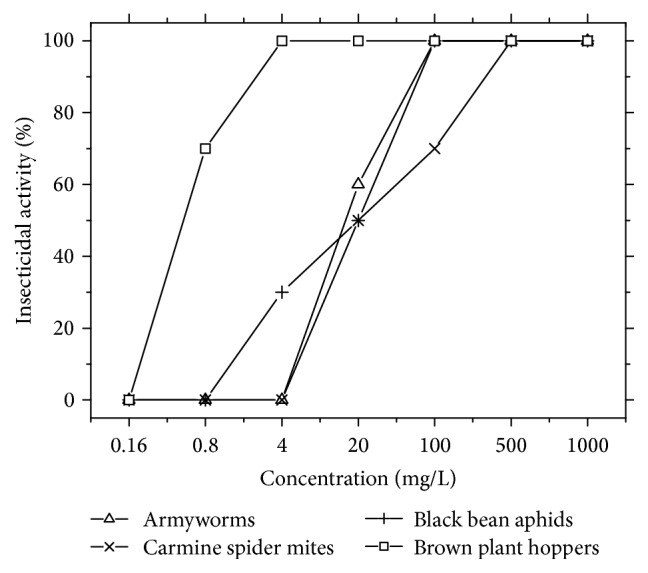
Insecticidal activity of avermectin against four kinds of pests.

Armyworms host more than 16 families and 104 species of plants, including cereal crops like wheat, rice, millet, and corn and other plants like cotton, beans, vegetables, and so on. Their larvae feed on leaves and cause serious damage. Because of their clustering, omnivory, and overeating, armyworms have become an important agricultural pest. The insecticidal property against armyworms was carried out by leaf dipping method. According to the results in Figure 5, the ability of killing armyworms increased with the concentration rising; when the concentration was 20 mg/L, this still showed killing capacity. Insecticidal property was weaker than avermectin technical material at concentration of 100 mg/L or less, but much stronger than carboxymethyl chitosan.

The insecticidal abilities against carmine spider mites, black bean aphids, and brown plant hoppers were investigated by spray method. Carmine spider mites are widely distributed in the temperate zone and are harmful to more than 32 families and 113 species of plants, including tomatoes, peppers, melons, beans, and onions. Black bean aphids and brown plant hoppers are primary pests of leguminous grass and rice, respectively. Both of them are perniciousness pests in agriculture. According to the results showed in Table 1, the insecticidal ability increased with the concentration rising. When the concentration was 500 mg/L, the lethal ratio was as much as 100%. The insecticidal property against the three pests showed a sequence of carmine spider mites > brown plant hoppers > black bean aphids. It showed a good insecticidal activity against black bean aphids and brown plant hoppers at a concentration as low as 4 mg/L. However, compared with the avermectin technical material, the insecticidal property of the conjugate was much weaker, especially at the concentration lower than 4 mg/L. This might be due to the content of avermectin in the conjugate.

According to the bioassay results above, it was discovered that the avermectin-grafted-NOCC conjugate showed enhanced insecticidal capacity compared with carboxymethyl chitosan. The conjugation of avermectin and carboxymethyl chitosan showed a new way to achieve insecticidal chitosan derivatives.

### 3.4. Photostability of Avermectin-Grafted-NOCC

**Figure 6:**
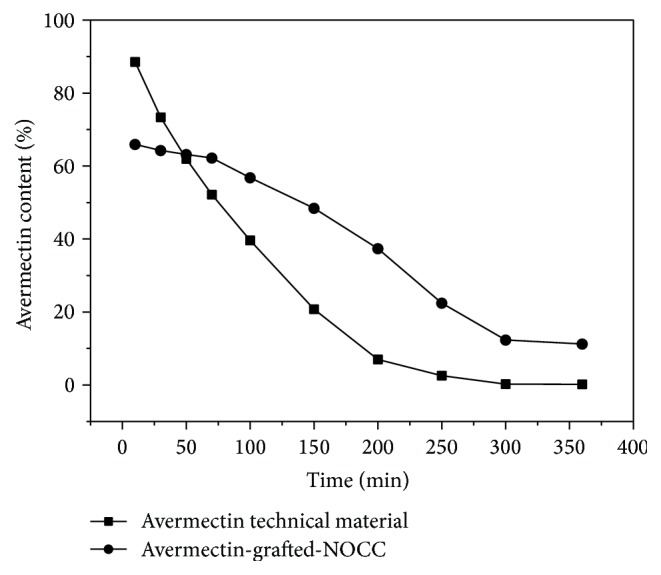
Photostabilities of avermectin-g-NOCC and avermectin technical material under UV-light.

The photostability of avermectin-grafted-NOCC was investigated in aqueous solution and UV-light. The results in Figure 6 showed enhanced photostability of the conjugate compared with the avermectin technical material. At 300 mins, the residual rate of the conjugate was 11.22%, while the residual rate of the avermectin technical material was just 0.2%. Between 300 mins to 360 min, the degradation process was also slowed down for the conjugate.

The possible mechanism of the photostability enhancement may be discussed in two parts. There are unsaturated groups in avermectin molecule, which absorb light easily [25]. In aqueous solution, the conjugates form self-assembled nanoparticles. The unsubstituted carboxymethyl group form the hydrophilic layer in the surface [26]. Thus, the substituted avermectin is protected from the light in such molecular structure. What is more, the photodegradation of avermectin is a series of chemical reactions initiated by oxidation reaction [27]. Carboxymethyl chitosan is a kind of antioxidant which captures the hydroxyl radical in the aqueous solution [28]. In this way, the inhibition of oxidation reaction can reduce the photodegradation of avermectin [29].

## 4. Conclusion

In our study, avermectin-grafted-NOCC was prepared successfully. The characterization and insecticidal activity of the new compound were investigated. The research showed that conjugation of avermectin and NOCC could be conducted by using DCC and DMAP. The formation of ester linkages was at the hydroxyl of avermectin and carboxyl groups of NOCC. Activity study indicated that the insecticidal activity of carboxymethyl chitosan was greatly improved by the conjugation with avermectin. Our research provided a new approach of the derivatization of chitosan. The avermectin-grafted-NOCC showed potential to be developed as novel insecticidal agents.

## Figures and Tables

**Table 1:** Insecticidal activity of avermectin against four kinds of pests.

Targets	Concentration (mg/L)	Insecticidal activity (%)		
		Avermectin-grafted-NOCC	Avermectin	NOCC
Armyworms	1000	100	100	0
	500	100	100	0
	100	100	100	0
	20	60	100	0
	4	0	100	0
	0.8	0	80	0
	0.16	0	0	0

Carmine spider mites	1000	100	100	0
	500	100	100	0
	100	70	100	0
	20	50	100	0
	4	0	80	0
	0.8	0	0	0
	0.16	0	0	0

Black bean aphids	1000	100	100	0
	500	100	100	0
	100	100	100	0
	20	50	100	0
	4	30	100	0
	0.8	0	0	0
	0.16	0	0	0

Brown plant hoppers	1000	100	100	0
	500	100	100	0
	100	100	100	0
	20	100	100	0
	4	70	100	0
	0.8	0	100	0
	0.16	0	90	0
